# In-gap corner states in core-shell polygonal quantum rings

**DOI:** 10.1038/srep40197

**Published:** 2017-01-10

**Authors:** Anna Sitek, Mugurel Ţolea, Marian Niţă, Llorenç Serra, Vidar Gudmundsson, Andrei Manolescu

**Affiliations:** 1 Science Institute, University of Iceland, Reykjavik, IS-107, Iceland; 2 School of Science and Engineering, Reykjavik University, Reykjavik, IS-101, Iceland; 3 Department of Theoretical Physics, Faculty of Fundamental Problems of Technology, Wroclaw University of Science and Technology, Wroclaw, 50-370, Poland; 4 National Institute of Materials Physics, Bucharest-Magurele, P.O. Box MG-7, Romania; 5 Institute of Interdisciplinary Physics and Complex Systems IFISC (CSIC-UIB), Palma de Mallorca, E-07122, Spain; 6 Department of Physics, University of the Balearic Islands, Palma de Mallorca, E-07122, Spain

## Abstract

We study Coulomb interacting electrons confined in polygonal quantum rings. We focus on the interplay of localization at the polygon corners and Coulomb repulsion. Remarkably, the Coulomb repulsion allows the formation of *in-gap states*, i.e., corner-localized states of electron pairs or clusters shifted to energies that were forbidden for non-interacting electrons, but below the energies of corner-side-localized states. We specify conditions allowing optical excitation to those states.

Core-shell quantum wires are vertically grown nanoscale structures consisting of a core which is covered by at least one layer of a different material (shell). Recently these structures attracted considerable attention as building blocks of quantum nanodevices^1^,^2^,^3^,^4^,^5^,^6^,^7^,^8^,^9^,^10^. A characteristic feature of core-shell systems is a non-uniform carrier distribution in different parts of the wire^11^,^12^,^13^,^14^,^15^,^16^,^17^. It is a consequence of the polygonal cross section which most commonly is hexagonal^18^,^19^,^20^, but may also be triangular^21^,^22^,^23^,^24^,^25^,^26^, square^27^, or dodecagonal^28^. Some of the properties of those wires, such as the band alignment^29^,^30^, may be controlled to a high extent. An appropriate combination of sample size and shell thickness allows to induce electron concentration on the shell area^18^. Moreover, the present technology allows for etching out the core part and producing nanotubes^19^,^20^.

Both nanowires and nanotubes may be viewed as polygonal quantum rings if they are sufficiently short, i.e., shorter than the electron wavelength in the growth direction. In this geometry the single-particle states with the lowest energy are localized in the corners of the polygon and are separated by a gap from the states localized on the sides. The gap can be of tens of meV or larger, depending on the shape of the polygon^31^,^32^.

The single-particle energy levels of a polygonal quantum ring are two- and fourfold degenerate and their arrangement is determined only by the number of polygon vertices. Similarly to the case of bent parts of quantum wires^33^,^34^,^35^,^36^,^37^,^38^,^39^,^40^, in the corner areas of polygonal quantum rings effective quantum wells are formed and thus low energy levels localize between internal and external boundaries. The number of such corner states is the number of vertices times two spin orientations. An energy gap may separate single-particle corner states from higher-energy states, the latter being distributed over the polygon sides^31^,^41^,^42^.

In this paper we extend the single-particle model of refs 31 and 32 to systems of few Coulomb interacting electrons. We show how this coupling allows the formation of states corresponding to electron pairs, or larger clusters, that localize on the corners and whose energies lie in the gap between corner and corner-side states of the uncoupled system. We focus on the formation and excitation of those many-body *in-gap states*, with particular emphasis on their fingerprints in optical absorption. As general motivations to study in-gap states in polygonal rings we mention their potential application in quantum information devices, exploiting the corner occupation as information unit, or their use as quantum simulators of discrete lattice models^43^.

## Results

Below we analyse systems of up to five electrons confined in triangular, square and hexagonal quantum rings. We take into account only conduction band electrons and neglect the valence levels or assume that the valence band is fully occupied. This situation can be achieved and controlled with a nearby metal gate, like in the single electron tunnelling experiments with quantum dots, or possibly with an STM tip.

For all of the analysed polygons the external radius (*R*_ext_) and side thickness (*d*) are fixed to 50 and 12 nm, respectively. The rings we describe are in fact short prismatic structures. We consider all electrons in the lowest mode in the growth direction, and the energy interval up to the second mode larger than the energy gap between the lateral modes of corner and side type. This gap varies from 27.5 meV for triangular rings to 4.1 meV for hexagonal samples. Assuming, e.g., the length (or height) in the growth direction equal to *d*, and the InAs parameters, an estimation of the energy separation between the two lowest longitudinal modes, using the simple quantum box model, gives 342 meV, which is above the energy range we are interested in this paper. In other words, the prism length is not important as long as it is short enough to guarantee sufficiently large separation between the two lowest longitudinal modes, i.e., larger than the energy range due to the lateral confinement.

### Many-body states for a triangular ring

**Figure 1:**
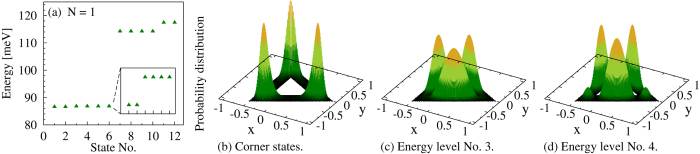
Single-particle quantities for a triangular ring. (**a**) The 12 lowest states arranged into 4 energy levels, the inset shows the degeneracy of corner states. (**b**–**d**) Probability distributions associated with the energy levels shown in Fig. (a). The *x* and *y* coordinates are in units of *R*_ext_.

The ground state of a single electron confined in a symmetric triangular quantum ring is twofold degenerate and is followed by a sequence of alternating pairs of four- and twofold degenerate levels, Fig. 1(a). For the analysed 12 nm thick ring the lowest six states are localized in the corners, with well-separated probability peaks in the areas between internal and external boundaries, and vanishing probability distributions on the sides of the ring, Fig. 1(b). The higher six states are distributed mostly over the sides of the triangle, with only little coverage of the corners, Fig. 1(c,d). In addition, corner and side states are separated by an energy gap Δ_*t*_ = 27.5 meV (*t* meaning *triangle*), which rapidly increases if the aspect ratio *d*/*R*_ext_ is reduced^31^,^32^.

**Figure 2:**
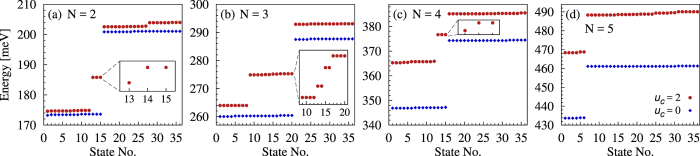
Energy levels for a triangular ring. The number of confined electrons (*N*) is shown in each figure and the interaction parameters shown in Fig. (**d**) are valid for all figures. In the insets to panels (**a**–**c**) we show the fine structure of the in-gap states.

**Figure 3:**
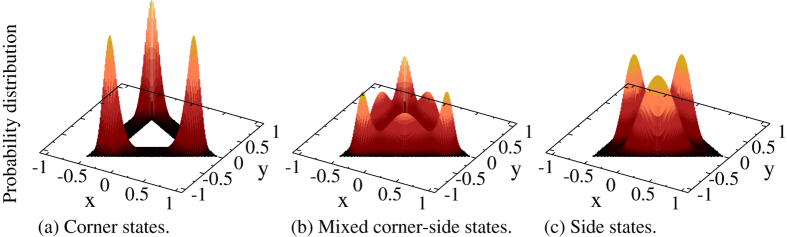
Two-particle lateral localization. Probability distributions for two electrons in corner (including the in-gap) states (**a**), in mixed corner-side states (**b**) and in side states (**c**). The *x* and *y* coordinates are in units of *R*_ext_.

For *N* = 2 non-interacting electrons confined in our triangular ring the low energy states form nearly dispersionless groups (flat bands), of fifteen corner states followed by thirty-six mixed corner-side states, represented by the blue diamonds in Fig. 2(a). Clearly, the two flat bands are separated by approximately Δ_*t*_. The lowest group of many-body states has probability distributions qualitatively similar to the single-particle corner-localized states, Fig. 3(a). The levels above the gap contain contributions from both, corner- and side-localized, single particle states and thus are associated with mixed corner-side probability distributions, Fig. 3(b). The third group of states is built up of only side-localized single particle states 7–12 in Fig. 1(a) and associated with probability distributions of that kind, Fig. 3(c).

The Coulomb interaction between the two electrons does not change qualitatively the charge distributions around the polygon shown in Fig. 3, as long as the Coulomb energy is smaller than Δ_*t*_. Instead, the energy spectrum differs qualitatively from the case of non-interacting particles, as shown in Fig. 2(a). The corner states 1–12 are only slightly shifted up, indicating that they correspond to electrons situated in different corners, in singlet or triplet spin configurations. The ground state is singlet and non-degenerate, the next energy levels are triplet sixfold, singlet twofold, and triplet threefold degenerate, respectively. These twelve states are only slightly spread within a narrow energy range, of about 0.25 meV.

Contrary to the behaviour of the first group of states, the next three corner states, 13–15, are shifted to higher energies, within the former gap of forbidden energies for non-interacting particles, Fig. 2(a). These in-gap states correspond to both electrons occupying the same corner area, with spin singlet configuration, and with an increased Coulomb energy. The localization of such states is still like in Fig. 3(a). Obviously, the charge density is equally distributed between the three symmetric corners.

The energy spectrum changes with the number of electrons. First of all it moves up due to the increased Coulomb energy. If *N* = 3 there are twenty many-body corner states, and twelve of them are lifted into the gap, Fig. 2(b). These states correspond to situations when two electrons of different spin occupy the same corner area while the third electron is localized around one of the two other corners. The energy spectrum for *N* = 4, Fig. 2(c), resembles the one for *N* = 2. This is a kind of particle-hole symmetry in the Fock space associated to the six single-particle corner states. In both cases three states are shifted into the gap, which for *N* = 4 correspond to two corners doubly occupied or one corner unoccupied. When *N* = 5, there are only six states associated with purely corner-localized probability distributions, with two corner areas occupied by a pair of electrons while the fifth electron stays on the third corner. As for *N* = 1, no in-gap state exists in this case, Fig. 2(d).

Degeneracies of the two-particle in-gap states, inset to Fig. 2(a), and the ones associated with one unoccupied corner area, inset to Fig. 2(c), reproduce the degeneracy of the lowest single-particle levels with respect to spin [inset to Fig. 1(a)] which in this case is conserved and thus the degeneracy of these levels is only of the orbital origin. This is not the case when some of the electrons are unpaired, such systems are spin polarized and some of their in-gap levels are fourfold degenerate, Fig. 2(b). In the presence of a magnetic field normal to the surface of the polygon the degeneracies are lifted. Still, the corner localization is not affected, as long as the Zeeman energy is smaller than the energy gap (Δ_*t*_), such that the mixing of corner and corner-side states is not significant.

### Comparison with the Hubbard model

The corner localization of the low-energy states suggests that we can obtain some insight from a Hubbard model with on-site Coulomb energy *U* and inter-site hopping energy *t*. Nevertheless, even for such a simplified model, only the simplest case of two electrons in triangular ring can be solved analytically^44^. The solution consists of 9 triplet states insensitive to interaction and 6 singlet states. Two singlets are non-degenerate while other two are both twofold degenerate, with energies


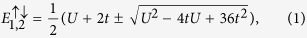



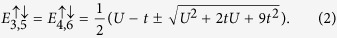


One can notice that in the limit of *U* ≫|*t*| the energies 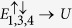, whereas 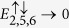. At the same time all triplet states have by default zero energy. This spectrum is qualitatively similar to the energies obtained earlier for the corner states of the thin triangle^32^. Therefore the energy difference between the in-gap states and the ground state is approximately the *U* parameter of the Hubbard model. The fine structure, i.e., splitting of the three in-gap states, is 
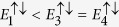
, for *t* < 0, in agreement with the previous results.

For three electrons on the triangle numerical results within the Hubbard model confirm twelve states shifted up by the Coulomb repulsion. However, for *U* ≫ |*t*|, their fine structure consists of three degenerate levels (4 + 4 + 4) instead of four (4 + 2 + 2 + 4) shown in Fig. 2(b).

A simple connection between the main model and the Hubbard one can be made if we keep in mind that, for a triangle with side thickness *d*, there is a simple relation between the energy of the in-gap states and the pair Coulomb energy *u*_*c*_. The in-gap states have an extra energy *E*_*c*_ = *u*_*c*_/*λ*, where *λ* is the distance between the two electrons sitting on the same corner, in units of *R*_ext_. Since the wave functions must vanish at the lateral boundaries *λ* should be smaller than *d*. From the energy data we estimate *λ* = 0.6*d*. Clearly *E*_*c*_ is linear with *u*_*c*_. From the results of the Hubbard model, if we assume no hopping between corners (*t* = 0), then we have *U* = *E*_*c*_.

### Many-body states for square and hexagonal rings

**Figure 4:**
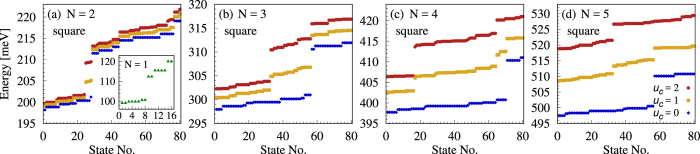
Energy levels for a square ring. The number of confined electrons (*N*) is shown in each figure and the interaction parameters given in Fig. d are valid for all figures.

The single-particle energy gap between corner and side states decreases with increasing number of corners^31^. For a 12 nm thick square ring this gap splitting is still sizeable, Δ_*s*_ = 11.8 meV (*s* meaning *square*), as shown in Fig. 4, but smaller than in the case of 12 nm thick triangle. As a result, the range of Coulomb interaction strengths *u*_*c*_ allowing formation of in-gap states reduces. For example, in Fig. 4 we show results for *u*_*c*_ = 1 and *u*_*c*_ = 2. For *N* = 2 four in-gap states are created, again corresponding to spin-singlet pairs occupying the same corner. The degeneracy of these states is 1, 2, 1 (in energy order). As for the triangle occupied by 2 or 4 electrons, they reproduce the degeneracy of the single particle corner states up to a spin factor of two. The spectra become more complex with increasing the number of electrons. Still, interesting effects occur, for example for *N* = 4. In this case two groups of in-gap states may be formed. One group with one singlet pair at one corner and the other two electrons in different corners, and another group, with a higher energy, but still in the gap, with two pairs at two corners. This situation is shown in Fig. 4(c) for *u*_*c*_ = 1. In the case of *N* = 5 only one group of in-gap states is formed, Fig. 4(d). When the interaction strength is increased to *u*_*c*_ = 2 then all states corresponding to two corner areas occupied by a singlet pair are shifted to energies above the gap and mix up with levels associated with corner-side-localized probability distributions [red circles in Fig. 4(c,d)].

**Figure 5:**
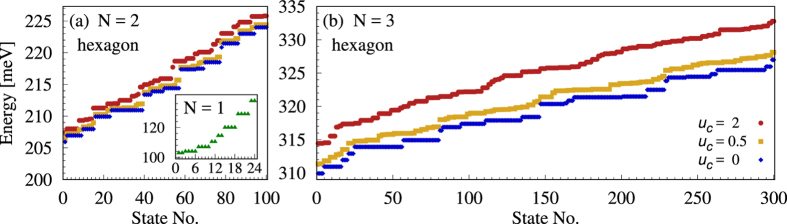
Energy levels for a hexagonal ring. The number of confined electrons (*N*) is shown in each figure and the interaction parameters given in Fig. b are valid for both figures.

For a hexagonal ring of 12 nm thickness the energy interval between the single-particle corner and side states is Δ_*h*_ = 4.1 meV, which is comparable to the energy spacing within these two groups of states. The reason is that the localization of the electrons is much weaker for the corner angle of 120 deg than for the previous cases of 60 and 90 deg. This results in the overlap of the energy domains of purely corner and mixed corner-side many-body states even for non-interacting particles. Consequently there is no energy gap above the many-body corner states in the examples shown in Fig. 5. The Coulomb interaction does not affect considerably the energy structure of such samples, it only shifts the levels to higher energies and changes the order of some states. However, the single-particle energy gap Δ_*h*_ increases with decreasing aspect ratio^32^, and thus, for sufficiently thin rings many-body corner states could be energetically separated from the other states as in the case of triangular and square samples. For such rings the Coulomb interaction either mixes higher corner and corner-side states or, if it is sufficiently weak, it reorganizes the corner states into groups corresponding to each number of close-by singlet pairs and different spatial separations of the particles. In all those cases in-gap levels cannot be identified as before. Still, theoretically, with a very low aspect ratio and a weak interaction such states could be obtained.

### Electromagnetic absorption

A single electron confined in a triangular quantum ring simultaneously exposed to a static magnetic field and circularly polarized electromagnetic field may be excited from its ground state to only four higher states within the 12 lowest states. Two of these states are associated with corner-localized probability distributions and originate from splitting of the second level of the degenerate system (*B* = 0) while the other two states belong to the group of side-localized states and merge into the third (fourfold degenerate) level when the magnetic field is removed. Two transitions, one to a state below and the other one to a state above the gap separating corner- from side-localized states, take place in the presence of each polarization type^31^,^32^.

**Figure 6:**
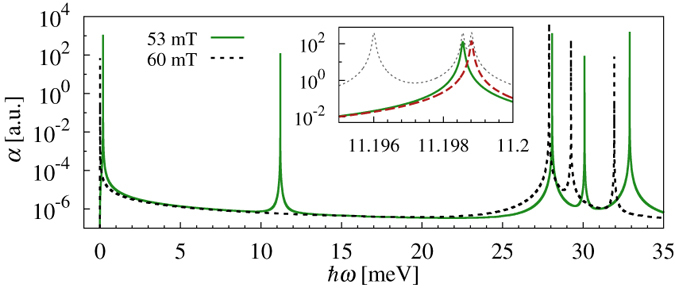
Absorption spectrum. Absorption coefficients associated with the excitation of the ground state of a pair of interacting electrons confined on a triangle impinged with clockwise polarized electromagnetic field and exposed to a magnetic field of 53 and 60 mT, close to the singlet-triplet ground state transition. Inset: Density of states (grey dotted) and absorption coefficients associated with clockwise (green solid) and counter-clockwise (red dashed) polarized electromagnetic field for the in-gap states.

Considering now a pair of Coulomb interacting electrons in the ground state (and *u*_*c*_ = 2), it may be excited with clockwise polarized electromagnetic field to one of the corner states with nearby energy, to one of the in-gap corner states, or to three states associated with mixed corner-side probability distributions (green solid lines in Fig. 6). One of the reasons why so many transitions are forbidden is that we do not take into account spin-orbit interaction and thus restrict transitions to pairs of states associated with the same spin. Moreover, some single-particle transitions are blocked due to wave function symmetry^31^,^32^. Consequently, the few allowed many-body transitions are the ones for which the corresponding dipole moment matrix elements contain the appropriate combinations of pairs of optically accessible single-particle states. Similar transitions to those shown in Fig. 6, but to different states, take place when the sample is excited with counter-clockwise polarized electromagnetic field.

In particular, in the inset to Fig. 6 we show the part of many-body absorption spectrum and density of states associated with the in-gap states for *B* = 53 mT. Two out of these states may be optically reached from the ground state, each one when the sample is impinged with differently polarized electromagnetic field (green solid and red dashed lines). The two final in-gap states are spin singlets, but split by the orbital effect of the magnetic field, and thus the transitions induced with clockwise and counter-clockwise polarization merge when the magnetic field is removed (*B* = 0).

**Figure 7:**
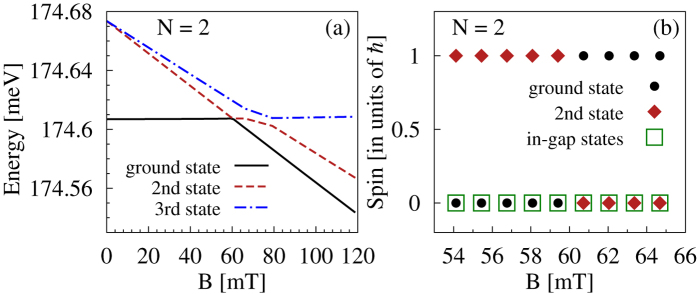
Effect of a magnetic field. (**a**) The three lowest energy states and (**b**) spin associated with the ground state, the first excited state, and the in-gap states of two electrons confined in a triangular ring versus magnetic field.

These in-gap states may be optically excited from the ground state only for a sufficiently low magnetic field, as long as the ground state is spin singlet, as it is for *B* = 0. In our case this regime corresponds to *B* < 60 mT. This situation changes when the external field reaches 60 mT. At this point a spin polarized state becomes the ground state, originating in a spin triplet at *B* = 0, as shown in Fig. 7(a). As seen in Fig. 7(b) the two lowest states are associated with different spin, so when the levels cross the spin of the ground state changes, while the in-gap states remain spin singlets over the transition range [green squares in Fig. 7(b)]. Consequently, the matrix elements of the dipole moment between the *new* ground state and the in-gap states vanish, together with the optical coupling, as indicated by the black dashed line in Fig. 6. The in-gap states may still be optically excited in the presence of higher magnetic fields, but from different initial states. Such states evolve from the ground state at *B* = 0, e.g. the second state for 60 < *B* < 80 T, or the third state for *B* > 80 mT [red dashed or blue dash-dotted lines in Fig. 7(b), respectively], or possibly from other spin singlet states.

## Discussion

We studied energy levels, localization, and optical absorption of systems of few electrons confined in polygonal quantum rings. If the numbers of corners and particles allow formation of purely corner-localized states corresponding to close-by singlet pairs, then the states associated with particular number of such pairs form separated groups which are shifted to higher energies and either form in-gap states in the energy ranges forbidden to non-interacting electrons, or mix up with levels associated with corner-side-localized probability distributions. An applied magnetic field (in the range of tens of mT) may lead to ground state spin change, for instance for two electrons towards spin alignment, while the in-gap states remain singlets, which results in blocking of the excitations towards the in-gap states. This provides a possibility of experimental testing of the sample shape and interaction strength with a contactless control of the absorption process. The presence of spin-orbit interaction in the core-shell structure, which would remove the spin selection rules, can also be experimentally tested. In general such a structure has a non-uniform dielectric constant, and thus the effective Coulomb potential is different from the standard 1/*r* form used in our model. Still, as long as the pairwise Coulomb energy is smaller than the gap between corner and side states our results remain qualitatively valid.

## Methods

**Figure 8:**
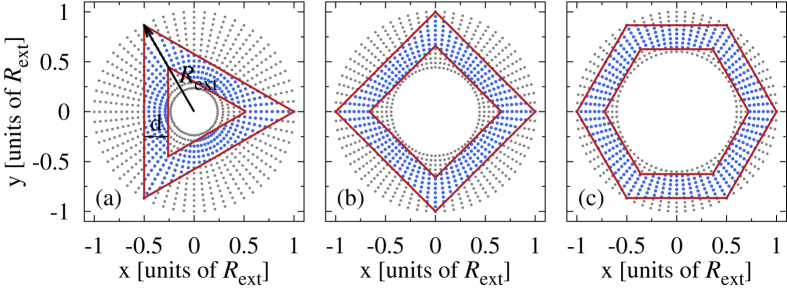
Sample models: Different polygonal constraints (red solid lines) applied on a polar grid (grey points) which is further reduced to sites situated only between the boundaries (blue points). The black arrows indicate the external radius of the polar grid and thus of the polygonal rings (*R*_ext_) and side thickness (*d*). For visibility we reduced the number of site points.

Our model of a polygonal quantum ring is based on a discrete polar grid^45^ on which we superimpose polygonal constraints and restrict to sites situated between the boundaries (Fig. 8). The single-particle Hamiltonian is


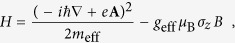


where ***A*** is the vector potential of an external magnetic field *B* normal to the ring plane (*x, y*), *m*_eff_ the effective mass of the ring material, *g*_eff_ the effective g-factor and *σ*_*z*_ the *z*th Pauli matrix. The Hilbert space associated with our polar lattice is spanned by the vectors |*kjσ*〉, including the radial (*k*) and angular (*j*) coordinates, and the spin (*σ*). The matrix elements 〈*kjσ*|*H*|*k*′*j*′*σ*′〉 are used to obtain single-particle eigenvalues *E*_*a*_ and eigenvectors *ψ*_*a*_ by numerical diagonalization^31^,^45^.

The many-body Hamiltonian of interacting electrons is


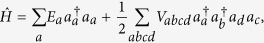


where operators 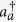 and *a*_*a*_ create and annihilate, respectively, an electron in the single-particle eigenstates, while *V*_*abcd*_ are the Coulomb integrals,


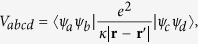


where *κ* is the material dielectric constant and |***r*** − ***r***′| the spatial separation of an electron pair. The many-body sates are obtained by (exact) diagonalization of 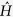 in a truncated Fock space, typically including a number of single particle states up to four times the number of polygon corners, and several thousands of grid points.

We calculate the absorption coefficients for the many-body system in the dipole and low temperature approximations. As derived e.g. in ref. 46, they are


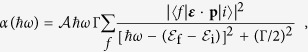


where 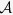 is a constant amplitude, 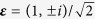 correspond to circular polarizations of the electromagnetic field, ***p*** is the electric dipole moment, Γ a phenomenological broadening, and 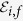 are the energies corresponding to the initial (|*i*〉) and final (|*f*〉) many-body states.

Our results were obtained for triangular, square and hexagonal quantum rings. For all of the analysed polygons the external radius (*R*_ext_) and side thickness (*d*) are fixed to 50 and 12 nm, respectively. In the numerical calculations we use as energy unit 
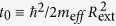
. Typically, we consider InAs as reference material, with *m*_eff_ = 0.023, *g*_eff_ = −14.9, and *κ* = 15. The strength of the Coulomb interaction of an electron pair is defined by the parameter *u*_*c*_ = (*e*^2^/*κR*_ext_)/*t*_0_, which is about 2.9 for In As. In our calculations we consider *u*_*c*_ = 1, 0.5 and 2. In order to resolve the fine structure of the absorption spectra we use Γ = 0.066 meV.

## Additional Information

**How to cite this article**: Sitek, A. *et al*. In-gap corner states in core-shell polygonal quantum rings. *Sci. Rep.* **7**, 40197; doi: 10.1038/srep40197 (2017).

**Publisher's note:** Springer Nature remains neutral with regard to jurisdictional claims in published maps and institutional affiliations.
